# The potential global distribution of *Chilo partellus*, including consideration of irrigation and cropping patterns

**DOI:** 10.1007/s10340-016-0801-4

**Published:** 2016-08-04

**Authors:** Tania Yonow, Darren J. Kriticos, Noboru Ota, Johnnie Van Den Berg, William D. Hutchison

**Affiliations:** 1 HarvestChoice, InSTePP, University of Minnesota, St. Paul, MN 55108 USA; 2 CSIRO, GPO Box 1700, Canberra, ACT 2600 Australia; 3 CSIRO, Private Bag 5, Wembley, WA 6913 Australia; 4 Unit for Environmental Sciences and Management, North-West University, Potchefstroom, 2520 South Africa; 5 Department of Entomology, Harvest Choice, University of Minnesota, St. Paul, MN 55108 USA

**Keywords:** CLIMEX, Niche modelling, Maize, Pest risk analysis, Sorghum, Spotted stem borer

## Abstract

*Chilo partellus* is a major crop pest in Asia and Africa, and has recently spread to the Mediterranean region. Knowledge of its potential distribution can inform biosecurity policies aimed at limiting its further spread and efforts to reduce its impact in areas that are already invaded. Three models of the potential distribution of this insect have been published, each with significant shortcomings. We re-parameterized an existing CLIMEX model to address some parameter inconsistencies and to improve the fit to the known distribution of *C. partellus*. The resulting model fits the known distribution better than previous models, highlights additional risks in equatorial regions and reduces modelled risks in wet and extremely dry regions. We bring new insights into the role of irrigation in the potential spread of this invasive insect and compare its potential distribution with the present known distribution of its hosts. We also distinguish regions that are suitable for supporting persistent populations from those that may be at risk from ephemeral populations during favourable seasons. We present one of the first demonstrations of a new capability in CLIMEX to automatically estimate parameter sensitivity and model uncertainty. Our CLIMEX model highlights the substantial invasion risk posed by *C. partellus* to cropping regions in the Americas, Australia, China, Europe, New Zealand and West Africa. Its broad host range and reported impacts suggest that it should be a pest of significant concern to biosecurity agencies in these presently uninvaded regions.

## Key message

- We fit a new CLIMEX model to improve our understanding of the factors limiting this pest’s distribution.
- This model addresses and resolves issues found in all previous models.
- Irrigation has a significant impact on the potential distribution of this pest.
- The potential distribution and risk of this pest is significantly larger than its current distribution.
- All areas where host crops are currently grown are at risk of attack, either from permanent populations, or from seasonal incursions.

## Introduction

**Fig. 1:**
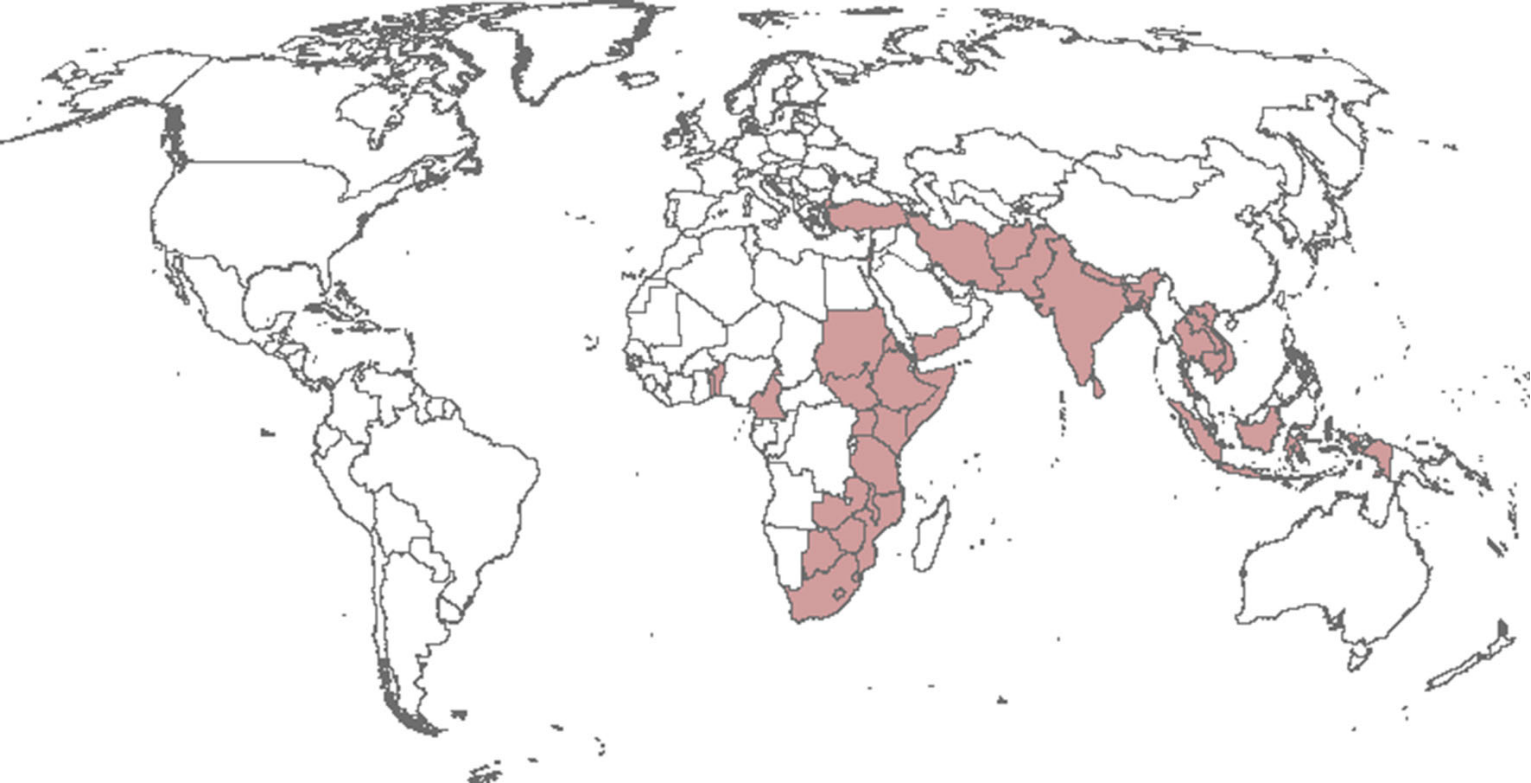
Map of the world, with shaded areas indicating those countries in which *Chilo partellus* has been previously recorded

*Chilo partellus* (Swinhoe) (Lepidoptera: Crambidae), the spotted stem borer, is possibly the most serious pest of maize and sorghum in eastern and southern Africa (e.g. Bate et al. 1991; Getu et al. 2001; Guofa et al. 2001; Harris 1990; Sylvain et al. 2015; Van den Berg et al. 1991) and a serious pest of maize and sorghum in Asia (e.g. Ahad et al. 2008; Ashfaq and Farooq-Ahmad 2002; Carl 1962; Dang and Doharey 1971; Harris 1990). It has also been noted to be a pest of sugarcane (Assefa et al. 2010; Carl 1962; Harris 1990), rice (Harris 1990) and pearl millet (Harris 1990). The species originates from Asia (Harris 1990; Kfir 1988), though its known distribution there appears poorly understood, with relatively few point location records available. Its distribution in Asia now includes Afghanistan, Bangladesh, Cambodia, India, Indonesia, Iran, Laos, Nepal, and Pakistan, Sri Lanka, Thailand, Vietnam and Yemen (Harris 1990; Rajabalee 1990, CABI Invasive Species Compendium datasheet 12859). In Africa, *C. partellus* was first reported in Malawi in 1930 (Tams 1932), and has since spread to Botswana, the Comoros Islands, Eritrea, Ethiopia, Kenya, Lesotho, Malawi, Mozambique, Somalia, South Africa, Sudan, Swaziland, Tanzania, Uganda, Zambia and Zimbabwe (Kfir et al. 2002; Overholt et al. 2000; Sylvain et al. 2015). It has been recorded from both Cameroon and Togo in West Africa (IAPSC 1985 in Harris 1990), and possibly occurs in Benin (GBIF data portal search shows 14 records for Benin in April 2014, from an animal census); however, the West African results remain unconfirmed, and are likely to be misidentifications (Overholt et al. 2000). More recently, *C.* *partellus* has been reported from the relatively dry regions of the Mediterranean Basin in Turkey (Bayram and Tonğa 2015) and Israel (Ben-Yakir et al. 2013). Figure 1 indicates the countries from which *C. partellus* has been previously recorded.

*Chilo partellus* has been rapidly expanding its range in Africa, from warmer lowlands into higher altitude regions (Guofa et al. 2001; Kfir 1993), displacing native stem borers (*Busseola fusca* and *Chilo orichalcociliellus*) of maize and sorghum (Kfir 1997a, b; Kfir et al. 2002). Putative reasons for its rapid spread and competitive abilities include a three-week shorter life cycle and a one month earlier termination of diapause compared with *B. fusca* (Dejen et al. 2014; Kfir 1997a).

As a result of the reported significant impacts of *C. partellus*, there have been several attempts to estimate its potential distribution: Overholt et al. (2000) used a GIS model, Hutchison et al. (2008) used CLIMEX (Sutherst and Maywald 1985) and Khadioli et al. (2014) used ILCYM (Insect Life Cycle Modeling software version 3.0). In reviewing each of these models, we identified significant shortcomings. As acknowledged by the authors, the GIS model of Overholt et al. (2000) simultaneously under-estimates the known distribution in South Africa and overestimates it in adjacent Zimbabwe. The CLIMEX model of Hutchison et al. (2008) includes internally inconsistent parameters and estimates much of central India to be unsuitable, where *C. partellus* is widespread and is known to occur. The ILCYM model of Khadioli et al. (2014) precludes persistence in Botswana, Zimbabwe and South Africa, where *C.* *partellus* is known to occur. All three models estimate suitable climate in western Africa and suggest that it is only a matter of time before *C.* *partellus* spreads there.

We revise the Hutchison et al. (2008) CLIMEX model, refitting parameter values according to the available literature and to better fit the known distribution. We also examine the projected distribution of *C. partellus* with those areas where host crops are present, to assess the areas at risk from invasion by this pest.

## Materials and methods

### Location records

Using several web sources (www.latlong.net and Google Earth), a number of locations for Asia were geo-coded from the literature (Ahad et al. 2008; Ashfaq and Farooq-Ahmad 2002; Attique et al. 1980; Carl 1962; Verma and Jotwani 1983; Jalali and Singh 2001; Jalali et al. 2010; Mahadevan and Chelliah 1986; Mohyuddin and Attique 1978; Neupane et al. 1985). As none of these publications present geo-referenced location points, we geo-coded the points based on the place names provided. Thus, these points are not exact location records [e.g. sampling is unlikely to have been done at Delhi airport, which is the location we used to geo-code Delhi (Verma and Jotwani 1983)], but they should nonetheless be sufficiently representative of the sampling sites as none of them occurred in regions of extreme topographic relief that can lead to significant mismatches between climate stations and field sites.

For Africa, geo-referenced location records were provided by Johnnie Van den Berg (obtained from personal observations and from colleagues) and were geo-coded from the literature (Cugala and Omwega 2001; Getu et al. 2001; Matama-Kauma et al. 2008).

### Meteorological and cropping data

We use the CM10_1975H CliMond dataset (Kriticos et al. 2012), comprising 30-year averages centred on 1975 at 10′ spatial resolution of monthly values for daily minimum and maximum temperature (°C), relative humidity (%) at 09:00 and 15:00, and monthly rainfall total (mm), to fit parameter values under a natural rainfall scenario.

**Fig. 2:**
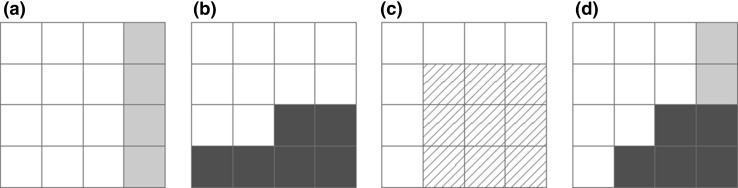
**a** *Shaded areas* are suitable under a natural rainfall scenario; **b** *shaded areas* are suitable under an irrigation scenario; **c** *hatched areas* are the irrigation areas identified by Siebert et al. (2005); **d** composite map of maximum EI values using shaded areas in both **a** and **b** considering the areas of irrigation in **c**

We apply an irrigation scenario of 2.5 mm day^−1^ throughout the year as top-up, to assess the risk posed by *C. partellus* in regions where cropping is sustained by irrigation. We use the irrigation areas identified by Siebert et al. (2005) to produce a composite map, comprising both irrigated and non-irrigated areas, to show the overall projected suitability for *C. partellus*. For each 10′ cell, if the irrigation area is greater than 0, the irrigation scenario result is used; otherwise, the natural rainfall scenario result is used (Fig. 2).

To assess the risk to agriculture, we mask this composite suitability map by the cropping areas for the various hosts of *C. partellus* (maize, sorghum, sugar cane, pearl millet and rice), using a union of the total area harvested from the two available versions of MapSPAM (Spatial Production Allocation Model) (You et al. 2012, 2014).

### Modelling strategy

We developed a new CLIMEX (Kriticos et al. 2015; Sutherst and Maywald 1985) model of the potential distribution of *C. partellus* (Table 1). We began with the Hutchison et al. (2008) parameter values, altering these according to the available literature (development, survival, reproduction and occurrence information) to provide a better fit to the known distribution in Asia under a natural rainfall scenario. A small number of location records did not fall within the projected potential distribution. Because these records appear to reflect populations that are able to persist only due to the use of irrigation to sustain cropping (e.g. Attique et al. 1980; Carl 1962), we ran the model with the irrigation scenario. As this resolved all issues with the Asian sites, we ran the model for Africa, first with the natural rainfall scenario and subsequently with the irrigation scenario, to see how well the resulting model accorded with the known distribution of *C. partellus*. Finally, 12 new location records for Africa were obtained (J. Van den Berg) and used to validate the model in relation to its performance in dry conditions. These location records all come from sites where more drought-tolerant sorghum is grown (Botswana, Zimbabwe, Lesotho and South Africa), or where maize is grown under irrigation (Namibia).

**Table 1:** CLIMEX parameter values for *Chilo partellus*

Parameters	Descriptions	Hutchison et al. (2008) values	Current values
Moisture			
SM0	Lower soil moisture threshold	0.1	0.1
SM1	Lower optimum soil moisture	0.8	0.8
SM2	Upper optimum soil moisture	0.95	**2**
SM3	Upper soil moisture threshold	1.25	**2.5**
Temperature			
DV0	Lower threshold	10 °C	**12** **°C**
DV1	Lower optimum temperature	25 °C	**27** **°C**
DV2	Upper optimum temperature	31 °C	**33** **°C**
DV3	Upper threshold	33 °C	**40** **°C**
Cold stress			
TTCS	Cold stress temperature threshold		
THCS	Temperature threshold stress accumulation rate		
DTCS	Degree-day cold stress threshold	15 °C-days^a^	15 °C-days
DHCS	Degree-day cold stress accumulation rate	−0.0001 week^−1^	−0.0001 week^−1^
Heat stress			
TTHS	Heat stress temperature threshold	33 °C	**40** **°C**
THHS	Temperature threshold stress accumulation rate	0.001 week^−1^	**0.01** **week** ^**−1**^
DTHS	Degree-day heat stress threshold		
DHHS	Degree-day heat stress accumulation rate		
Dry stress			
SMDS	Soil moisture dry stress threshold	0.2	**0.1**
HDS	Stress accumulation rate	−0.005 week^−1^	**−0.035** **week** ^**−1**^
Wet stress			
SMWS	Soil moisture wet stress threshold	2.5	2.5
HWS	Stress accumulation rate	0.002 week^−1^	**0.01** **week** ^**−1**^
Threshold heat sum			
PDD	Number of degree-days above DV0 needed to complete one generation	600 °C-days	**700** **°C-days**
Irrigation scenario	2.5 mm day^−1^ as top-up throughout the year		

Changes made to the Hutchison et al. (2008) parameter values are given in bold

^a^Hutchison et al. (2008) used 5 °C as the base temperature for the cold stress calculation, not DV0 = 10 °C

### Parameter adjustment

Table 1 lists all parameter values used in the model. To simplify the comparison of our model to that of Hutchison et al. (2008), we provide both sets of parameter values in the table. To minimise repetition in the text, we do not refer to Table 1 in each section below, where we address the changes made to the parameter values. Differences between our parameter values and those of Hutchison et al. (2008) are given in bold.

#### Moisture parameters

We increase both the upper optimum (SM2) and the upper threshold (SM3) parameters, to make the summer rainfall conditions in the Jammu region of India suitable for population growth (Ahad et al. 2008). The parameter values of Hutchison et al. (2008) model this area as too wet in July and August, when Ahad et al. (2008) show high adult trap catches and impose an incorrect bi-modal seasonality with two small peaks of growth in spring and autumn.

#### Temperature parameters

We increase all of the temperature parameters. Whilst Mbapila et al. (2002) and Khadioli et al. (2014) report *estimates* for the lower developmental threshold to be between 9 and 11 °C, no egg development or hatch was observed at either 13 or 15 °C (Jalali and Singh 2001; Khadioli et al. 2014), nor larval development at 13 °C (Jalali and Singh 2001). A value of 12 °C for DV0 is consistent with these data.

The lower and upper optimal temperatures are increased (DV1 = 27 °C and DV2 = 33 °C), to better span the range of optimal temperatures either observed or estimated by various authors. Mbapila et al. (2002) show that R_o_ peaks at 28–31 °C. Khadioli et al. (2014) show minimum mortality of eggs and larvae at 30 °C and of pupae at 32 °C, and calculates the optimum temperature for immature stages to be between 32 and 33 °C. These values for the optimum range are also consistent with results of other authors (Dang and Doharey 1971; Mahadevan and Chelliah 1986; Singh 1991; Tamiru et al. 2012).

The upper temperature threshold is increased from 33 °C to 40 °C. Full development does not occur at 37 °C (Jalali and Singh 2001), 38 °C (Khadioli et al. 2014) or 40 °C (Singh 1991); however, *C. partellus* occurs year-round at Hisar, in north-west India (Taneja and Leuschner 1985), with peak adult trapping occurring from August to October. This indicates that there is growth of immature stages earlier in the summer, when maximum temperatures exceed 40 °C. To allow for population growth to occur in June and July, during the favourable parts of the days when temperatures are lower than this, DV3 is increased to 40 °C.

#### Cold stress (CS)

We do not adjust the cold stress parameters, although we do ensure that the degree-day cold stress calculation uses the developmental temperature threshold (DV0). Thus, anyone entering our parameter values into a CLIMEX model will obtain the same results. These parameters result in most of Nepal being suitable for *C. partellus* (e.g. see Harris 1990; Neupane et al. 1985). Because DV0 is higher in our model than that of Hutchison et al. (2008), more CS accumulates in our model. However, the only location records of *C. partellus* that experience any CS are in South Africa, Lesotho and northern Pakistan, and the highest level of CS accumulated at any of these sites is only 22.

#### Heat stress (HS)

For internal consistency, because we increase the upper developmental threshold (DV3) to 40 °C, at the very least, we have to increase the HS temperature threshold (TTHS) to the same value. It is no longer acceptable practice in CLIMEX modelling to have stress accumulation occurring within the bounds set for population growth (Kriticos et al. 2005). This relationship between growth and stress parameters has been enforced within CLIMEX since version 3. It is only possible to over-ride this default set of relationships for backwards compatibility with older models. We use a threshold value of 40 °C and a reasonably high stress accumulation rate (0.01 week^−1^).

The HS parameter values (Table 1) provide low levels of HS in central India, but provide excessive HS in the border region of India and Pakistan, and further west into the central region of Pakistan.

#### Dry stress (DS)

The Hutchison et al. (2008) model has DS accumulating within the bounds set for growth, which is not acceptable practice in CLIMEX modelling (Kriticos et al. 2005). As we reduce SMDS to the growth threshold (SM0) of 0.1, we increase the rate of stress accumulation. Under a natural rainfall scenario, prohibitive DS occurs in much of western India and the southern half of Pakistan. However, as irrigation is used to grow the *rabi* (spring-harvested) crops (e.g. maize, sorghum, rice millets, soybean, groundnut), most of these areas become suitable under a top-up irrigation scenario. This DS rate also shows the maximum value that allows persistence in the Okavango panhandle in Botswana under a natural rainfall scenario.

#### Wet stress (WS)

In CLIMEX, WS limits the range of a species under conditions of excessive soil moisture. For an insect such as *C. partellus*, this may primarily be an effect on its hosts. The Hutchison et al. (2008) WS parameters contribute nothing to defining the potential range of *C.* *partellus.* We increase the stress accumulation rate to preclude extremely wet areas from being suitable. The change has no impact on the known distribution of *C. partellus*, but it does preclude persistence in areas receiving in excess of about 4 700 mm annual rainfall.

#### Degree-days per generation (PDD)

A powerful form of cross validation of CLIMEX models is the ability to compare the estimated number of generations with field reports. The literature was searched to identify locations and corresponding reports of the number of generations of *C. partellus*. For each of these locations, we extract the simulated number of generations from the CLIMEX model and compare the two datasets. The current value of 700 degree-days above 12 °C provides results that accord with information in the literature.

#### Parameter sensitivity and model uncertainty

A new option in CLIMEX Version 4 is the ability to undertake automated sensitivity and uncertainty analyses (Kriticos et al. 2015). The sensitivity analysis identifies the degree to which each species parameter affects the projected total area of suitable habitat and other model state variables. The result of the one-parameter-at-a-time sensitivity analysis is a table of values indicating relative sensitivity of each parameter for each state variable.

The closely related uncertainty analysis takes into account the fact that the parametric uncertainty affects all of the parameters simultaneously. Consequently, it uses a Latin hypercube to sample a triangular distribution of values spanning each of the default parameters used in the species parameter file. To address the anisotropism between variables, the uncertainty bounds are scaled differently for each parameter type. Therefore, the results reflect a general sense of uncertainty associated with our ability to estimate each of the parameter types, rather than any specific consideration of our confidence in our ability to estimate each specific parameter (Kriticos et al. 2015). The result of the uncertainty analysis is an agreement map indicating the proportion of models from the Latin hypercube sampling (*n* = 50) that resulted in a suitable Ecoclimatic Index value (EI > 0).

For each of the sensitivity and uncertainty analyses, the default model parameters were run using the same CM10 1975H V1.1 dataset used for the model fitting process. The analyses were only performed for the natural rainfall scenario. The results of both the sensitivity and uncertainty analyses depend on the region over which the analyses are run. In this case, the analyses were run for the entire world.

## Results

### Distribution

**Fig. 3:**
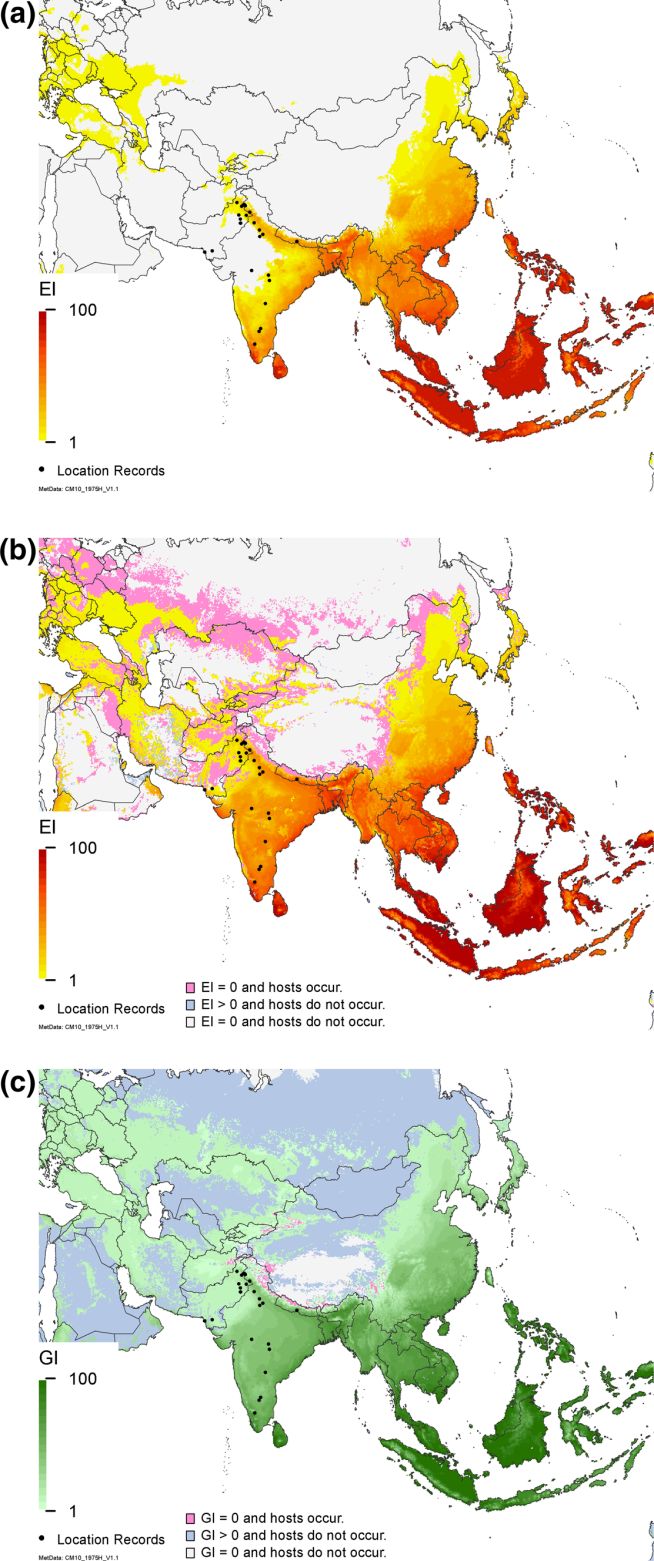
Modelled climate suitability of Asia for *Chilo partellus* **a** to persist as a permanent population under a natural rainfall scenario, **b** to persist as a permanent population mapped as a composite of natural rainfall and irrigation based on the irrigation areas identified by Siebert et al. (2005), then masked by harvested areas of host plants (maize, sorghum, sugarcane, pearl millet and rice) (You et al. 2012, 2014) and **c** to have positive growth in harvested areas of host plants under an irrigation scenario regardless of the potential to persist as a permanent population. Location records geo-coded from the literature (Ahad et al. 2008; Ashfaq and Farooq-Ahmad 2002; Attique et al. 1980; Carl 1962; Verma and Jotwani 1983; Jalali and Singh 2001; Jalali et al. 2010; Mahadevan and Chelliah 1986; Mohyuddin and Attique 1978; Neupane et al. 1985)

The modelled potential distribution in Asia encompasses the current known distribution. It distinguishes areas where *C. partellus* can occur under natural rainfall conditions (Fig. 3a) from those where it can persist only because irrigation is used in the dry season to sustain agriculture (Attique et al. 1980; Carl 1962) (Fig. 3b). The projected area of suitability extends beyond the current distribution, to include countries such as Japan, North and South Korea, China, Malaysia and the Philippines. As these countries are climatically suitable, hosts are present, and there are no apparent barriers to dispersal, they are likely at risk from invasion of *C. partellus*. Some current cropping regions appear not to be able to support permanent populations of this pest (Fig. 3b); however, we suggest that these areas are nonetheless at risk from seasonal incursions, as the annual growth index (GI_A_) is positive throughout these temperate cropping areas (Fig. 3c). Cropping apparently occurs in some areas of Asia where the GI_W_ for *C. partellus* is zero (Fig. 3c), but as these are high-altitude areas where the maximum temperatures rarely exceed 10 °C, it is difficult to see how crops could be grown here. These areas more likely represent errors in the cropping database (You et al. 2012, 2014).

**Fig. 4:**
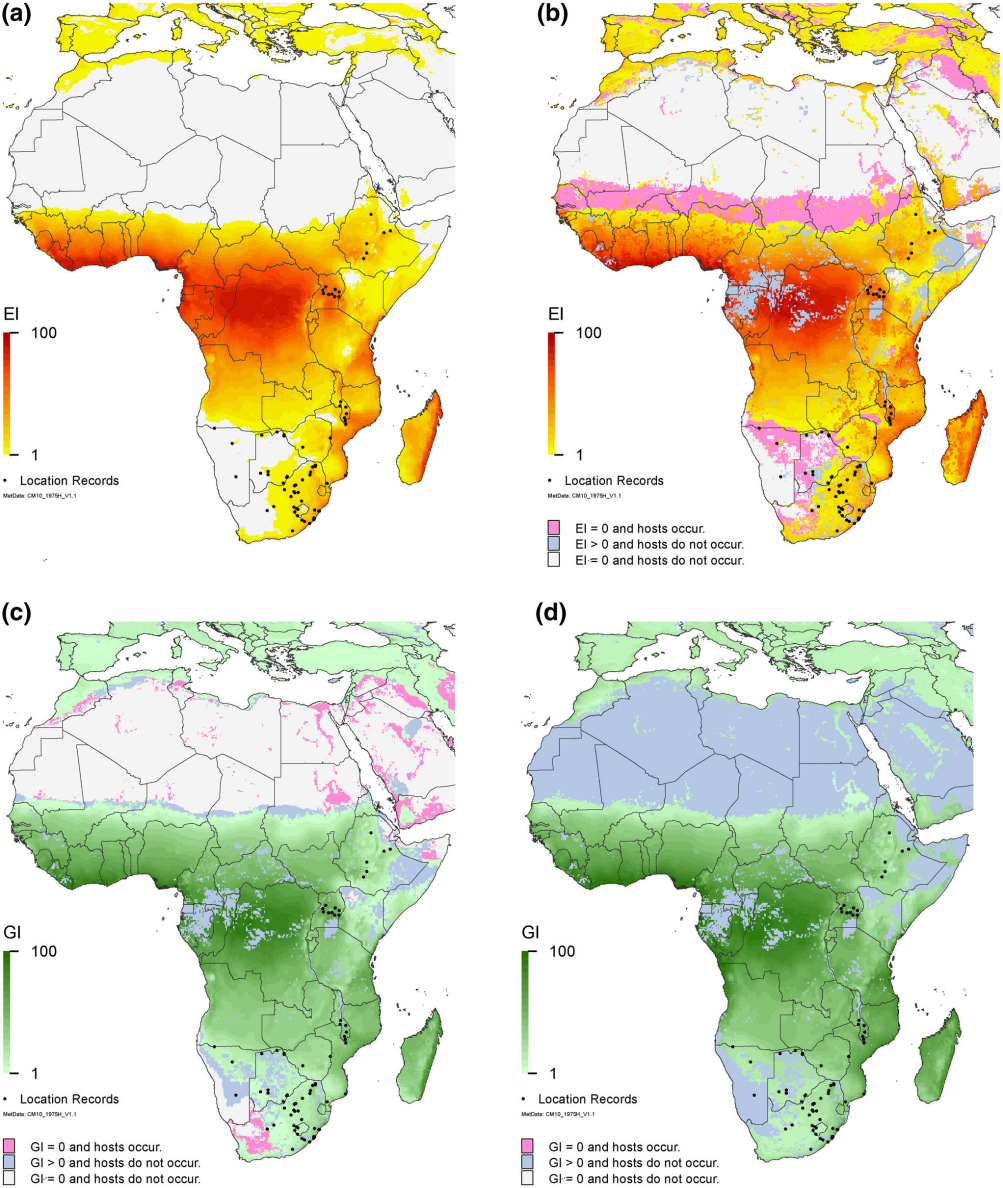
Modelled climate suitability of Africa for *Chilo partellus* **a** to persist as a permanent population under a natural rainfall scenario; **b** to persist as a permanent mapped as a composite of natural rainfall and irrigation based on the irrigation areas identified by Siebert et al. (2005), then masked by harvested areas of host plants (maize, sorghum, sugarcane, pearl millet and rice) (You et al. 2012, 2014); **c** to have positive growth in harvested areas of host plants under natural rainfall conditions regardless of the potential to persist as a permanent population and **d** to have positive growth in harvested areas of host plants under an irrigation scenario regardless of the potential to persist as a permanent population. Location records were provided by Johnnie Van den Berg from personal observations and colleagues, and were geo-coded from the literature (Cugala and Omwega 2001; Getu et al. 2001; Matama-Kauma et al. 2008)

In Africa, the projected potential distribution of *C. partellus* encompasses most location records without the use of irrigation: only four location records from our validation set are distinctly isolated from regions modelled as suitable for persistent occupation (Fig. 4a). All bar one locations (Hukuntsi, in Botswana) become suitable in the composite map (Fig. 4b), in agreement with our knowledge that these validation records are from areas where agriculture occurs with irrigation. We examined these locations in Google Earth and we found patterns suggesting sporadic cropping, lending further support for this conclusion. Given the model results, the projected range of *C. partellus* and the historical patterns of cropping, it appears that this pest could expand its range significantly, to potentially encompass all of central and much of western Africa. The composite host-masked suitability map (Fig. 4b) indicates that some agricultural areas are not at risk of *C. partellus* becoming permanently established; however, these areas can nonetheless support growth of transient (seasonal) populations migrating from suitable locations nearby (Fig. 4c, d).

The composite suitability map for Africa (Fig. 4b) shows that only one of the validation sites (Hukuntsi in Botswana) falls outside the projected range of suitability, with an Ecoclimatic Index (EI) of zero. This is because Siebert et al. (2005) do not include this area as a region of agriculture maintained with irrigation. Google Earth images also suggest that any cropping here depends upon rainfall: it is largely scrubland, and whilst there appear to be sparsely distributed clearly demarcated fields, there is no permanent source of water for irrigation. This area was sampled as positive for *C.* *partellus* in 2001 (J. Van den Berg, pers. comm.), hence it is possible that (a) there was a seasonal incursion into the area from nearby locations supporting permanent populations and (b) the sample was collected in a wetter than average year. It is entirely possible that this area experiences seasonal incursions of *C. partellus*, as it is not far from two locations that support permanent populations, and the entire region has a positive annual growth index (GI_A_) that would allow for some seasonal growth (Fig. 4c). To test whether or not 2001 was a wetter than average year, we ran the CLIMEX Compare Years/Locations module with the WFDEI dataset (Weedon et al. 2014) from 1990 to 2005, and whilst most of Botswana is generally not suitable (EI = 0), the years 2000 and 2001 show large parts of the country (including this area) as being suitable (EI ≥ 1). Similarly, whilst the annual growth index (GI_A_) maps show that seasonal growth is possible throughout Botswana in most years, there is a marked increase in the GI_A_ for these 2 years. Thus, although on average, this area is too dry for permanent populations of *C. partellus* to persist (Figs. 4a, b), seasonal growth (i.e. due to migration) is possible both under a natural rainfall scenario (Fig. 4c) and with irrigation (Fig. 4d). Furthermore, the positive sample appears to have been recorded in a wetter than average year.

**Fig. 5:**
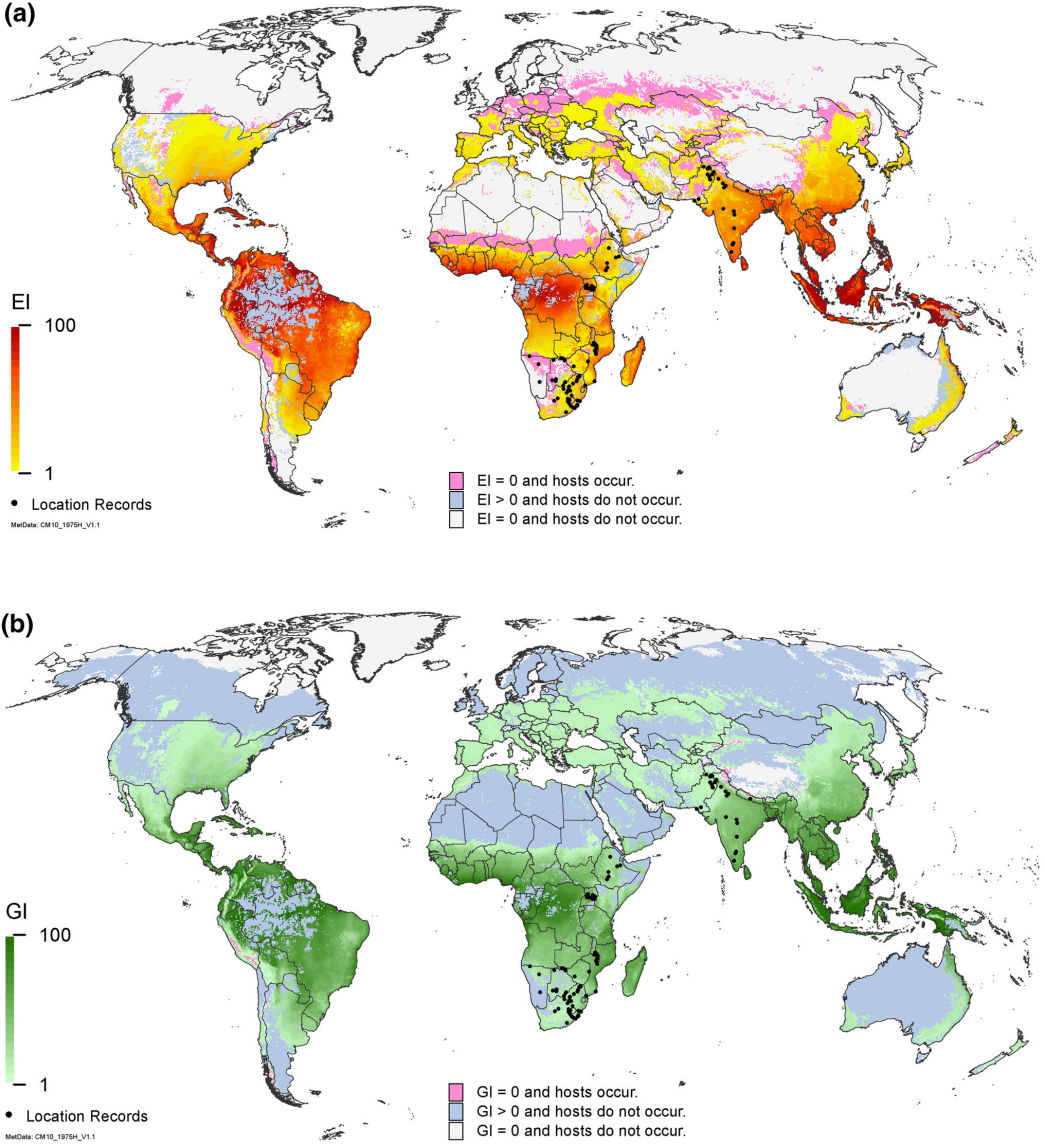
Modelled global climate suitability for *Chilo partellus* **a** to persist as a permanent population, mapped as a composite of natural rainfall and irrigation based on the irrigation areas identified by Siebert et al. (2005), then masked by harvested areas of host plants (maize, sorghum, sugarcane, pearl millet and rice) (You et al. 2012, 2014) and **b** to have positive growth in harvested areas of host plants under an irrigation scenario regardless of the potential to persist as a permanent population. Location records for Asia were geo-coded from the literature (Ahad et al. 2008; Ashfaq and Farooq-Ahmad 2002; Attique et al. 1980; Carl 1962; Verma and Jotwani 1983; Jalali and Singh 2001; Jalali et al. 2010; Mahadevan and Chelliah 1986; Mohyuddin and Attique 1978; Neupane et al. 1985). Location records for Africa were provided by Johnnie Van den Berg from personal observations and colleagues, and were geo-coded from the literature (Cugala and Omwega 2001; Getu et al. 2001; Matama-Kauma et al. 2008)

Globally (Fig. 5a), our results show that many of the agricultural areas where host crops are grown are at risk from *C. partellus*. Many of these regions have a high suitability (EI) index, suggesting that introductions into these areas could incur serious impacts if control efforts applied to other insect pests do not also control *C. partellus*. Cropping areas designated as unsuitable for the permanent establishment of *C. partellus* populations are potentially still at risk from seasonal invasions (Fig. 5b).

### Voltinism

The number of generations simulated accords well with the numbers reported in the literature (Table 2). Unfortunately, the literature does not provide much hard data with which to compare our model. As shown in Table 2, most of the references that mention a number of generations either calculate this from other estimates, or make statements without providing the supporting data. The current value of 700 °C-days used in our model provides the appropriate number of generations for Chitwan, Bangalore, Ludhiana, the Punjab region of Pakistan and Mozambique, and allows just over three generations to be completed at Potchefstroom. Van Rensburg and Van Den Berg (1992) suggests that there are possibly four overlapping generations of *C. partellus* in the Western Transvaal, but it is not clear whether or not the fourth generation is in fact completed. Our model indicates that more generations can be completed in Brits and Warmbaths than are reported, but it appears that *C. partellus* larvae undergo diapause at these locations (Kfir 1988, 1992), which will reduce the number of generations actually completed. Various authors (Atwal et al. 1969; Kfir 1992; Kfir et al. 2002; Neupane et al. 1985; Van Rensburg and Van den Berg 1992) indicate that overlapping generations occur, making it difficult to determine exactly how many generations are completed. Our model nonetheless indicates that the correct number of generations can be completed at all but two locations (Brits and Warmbaths).

**Table 2:** Comparison of reported and modelled number of generations of *Chilo partellus*

Location	Authors	Reported number of generations per year	Modelled number of generations per year	Number of degree-days >12 °C
Chitwan, Nepal	Neupane et al. (1985)	At least 5	6.29	3738
Bangalore, southern India	Jalali and Singh (2001)	6–7, calculated from estimated developmental thresholds for each life stage	6.21	3619
Ludhiana, north-west India	Atwal et al. (1969)	6	6.61	3994
Rawalpindi, Punjab region, Pakistan	Attique et al. (1980)	4–5 by August (no data to support statement)	5.42	3208
Yousafwala, Punjab region, Pakistan	Attique et al. (1980)	4–5 by August (no data to support statement)	6.78	4115
Mozambique	Sithole (in ICRISAT 1989) and Kfir et al. (2002), both citing Berger pers. comm. and Berger 1981	At least 3	3.80–7.58	2000–4600*
Brits, South Africa	Kfir (1992)	2.5	3.97	2206
Warmbaths, South Africa	Kfir (1992)	1.5	3.93	2172
Potchefstroom, South Africa	Van Rensburg and Van den Berg (1992)	3-4; not clear whether the 4th generation is completed or not	3.14	1664

* Degree-days for Mozambique were taken for grid cells showing the lowest and highest number of generations, and therefore the lowest and highest number of degree-days above 12 °C

### Phenology

**Fig. 6:**
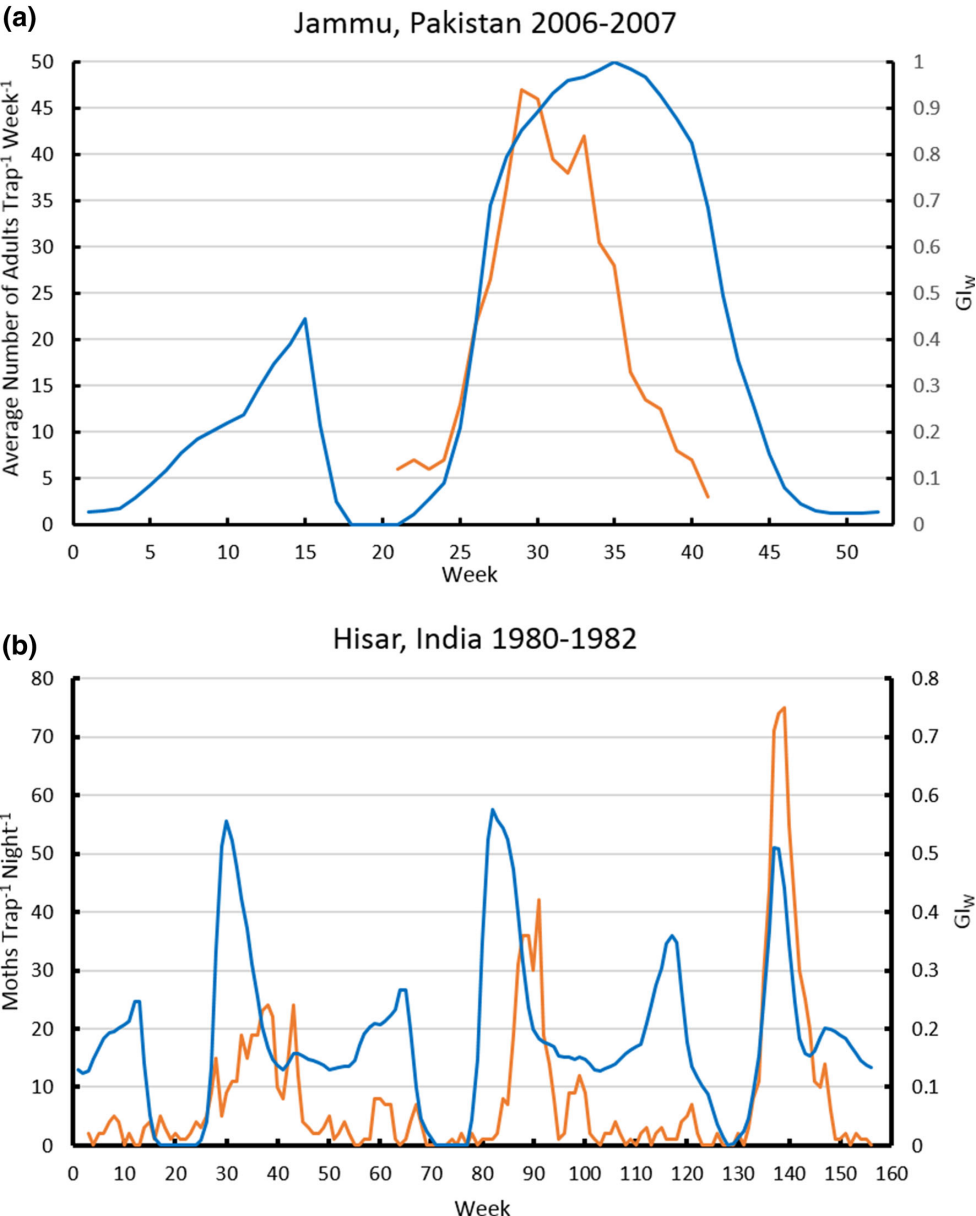
**a** Population dynamics of *Chilo partellus *in Jammu, Pakistan, showing trap catch data provided in Ahad et al. (2008) and the weekly growth index, averaged for 2006–2007, produced with the Compare Locations/Years module with the WFDEI dataset (Weedon et al. 2014) under a natural rainfall scenario. **b** Population dynamics of *Chilo partellus *in Hisar, north-western India, with trap catch data for 1980–1982 extracted from Fig. 1 in Taneja and Leuschner (1985) overlain on the weekly growth index for the same years, produced with the Compare Locations/Years module run on WFDEI dataset (Weedon et al. 2014) and using the top-up irrigation scenario. Trap catch data are in orange; GI_W_ is in blue

We compare modelled phenology to reported information, to confirm that the model correctly represents observed seasonality patterns. Summer rainfall patterns need to allow population growth and trapping of adults around Jammu (Ahad et al. 2008). Figure 6 shows that we are simulating very similar phenology for those particular years, with growth occurring from May to October.

Taneja and Leuschner (1985) report 3 years (1980–1982) of trap catches near Hisar, in northern India, showing that adults are trapped year-round, with the main peak in activity between August and October. As this area is very dry, we use the irrigation scenario for the analysis. The CLIMEX Compare Years/Locations run illustrates similar variability in the GI_W_ (Fig. 6b). CLIMEX indicates that peak population growth occurs earlier than the peak trap catches, corresponding with adult activity following larval development. Both of these analyses confirm that appropriate phenological patterns are being simulated by our model.

### Parameter sensitivity and model uncertainty

The parameter sensitivities are presented in Table 3. The model parameters are listed in descending sensitivity for modelled range. The dry stress threshold and the related minimum soil moisture level for population growth are the most sensitive parameters, with a 7.02 and 2.23 % impact, respectively. The range over which they have been tested 0–0.2 is relatively large, and the impact is relatively minor. Our confidence in the default value for SMDS and SM0 (0.1) is quite high. It accords with the approximate value for permanent wilting point, and results in a modelled range boundary accord with the distribution of *C. partellus* in xeric environments. The next most sensitive parameter is the minimum temperature for development (DV0, 1.64 % impact on modelled potential range). The experimental and other evidence used to support the selection of this parameter value suggests that the true value probably does lie within this range explored in the sensitivity analysis. The number of degree-days per generation (PDD) had a sensitivity of 1.44 %. Our confidence in this parameter is reasonable, given the concordance of the number of generations simulated and reported. The next most sensitive parameter is the dry stress accumulation rate (HDS), which has only 1.19 % sensitivity. This fitted value accords well with the known distribution of the species, considering the variability of climate and the existence of ephemeral cropping areas in xeric locations. The remainder of the parameters have 1 % or less sensitivity to the modelled potential range. The stress and growth variables are most sensitive to their corresponding parameters, which is a check of logically consistency.

**Table 3:** CLIMEX parameter sensitivity values for *Chilo partellus* parameters listed in Table 1, as applied to the CM10 1975H V1.1 global dataset under a natural rainfall scenario

Parameter	Mnemonic	Parameter values			Range change (%)	EI change	Core distribution change (%)	Growth variables			Stress variables			
		Low	Default	High				MI change	TI change	GI change	CS change	DS change	HS change	WS change
Dry stress threshold	SMDS	0	0.1	0.2	7.02	10.09	11.07	0	0	0	0	63.42	0	0
Limiting low moisture	SM0	0	0.1	0.2	2.23	2.11	0	11.09	0	2.9	0	0	0	0
Limiting low temperature	DV0	11	12	13	1.64	1.15	0	0	2.4	1.4	0	0	0	0
Degree-days per generation	PDD	560	700	840	1.44	0.39	0	0	0	0	0	0	0	0
Dry stress rate	HDS	−0.042	−0.035	−0.028	1.19	1.82	0.12	0	0	0	0	8.47	0	0
Cold stress degree-day rate	DHCS	−0.00012	−0.0001	−0.00008	0.79	0.79	0.04	0	0	0	11.47	0	0	0
Cold stress degree-day threshold	DTCS	14	15	16	0.32	0.4	0.1	0	0	0	5.4	0	0	0
Lower optimal moisture	SM1	0.7	0.8	0.9	0.32	1.41	0	7	0	1.8	0	0	0	0
Lower optimal temperature	DV1	26	27	28	0.15	2.93	0	0	4.7	3.1	0	0	0	0
Upper optimal temperature	DV2	32	33	34	0.02	0.65	0	0	3.3	0.9	0	0	0	0
Heat stress temperature threshold	TTHS	39	40	41	0.02	0.24	0	0	0	0	0	0	15.26	0
Limiting high temperature	DV3	39	40	41	0.01	0.13	0	0	2.5	0.2	0	0	0	0
Wet stress rate	HWS	0.008	0.01	0.012	0.01	0.15	0	0	0	0	0	0	0	0.6
Wet stress threshold	SMWS	2.4	2.5	2.6	0.01	0.25	0.03	0	0	0	0	0	0	0.8
Heat stress temperature rate	THHS	0.008	0.01	0.012	0	0.03	0	0	0	0	0	0	3	0
Upper optimal moisture	SM2	1.9	2	2.1	0	0.49	0	0.6	0	0.5	0	0	0	0
Limiting high moisture	SM3	2.4	2.5	2.6	0	0.3	0	0.37	0	0.3	0	0	0	0

**Fig. 7:**
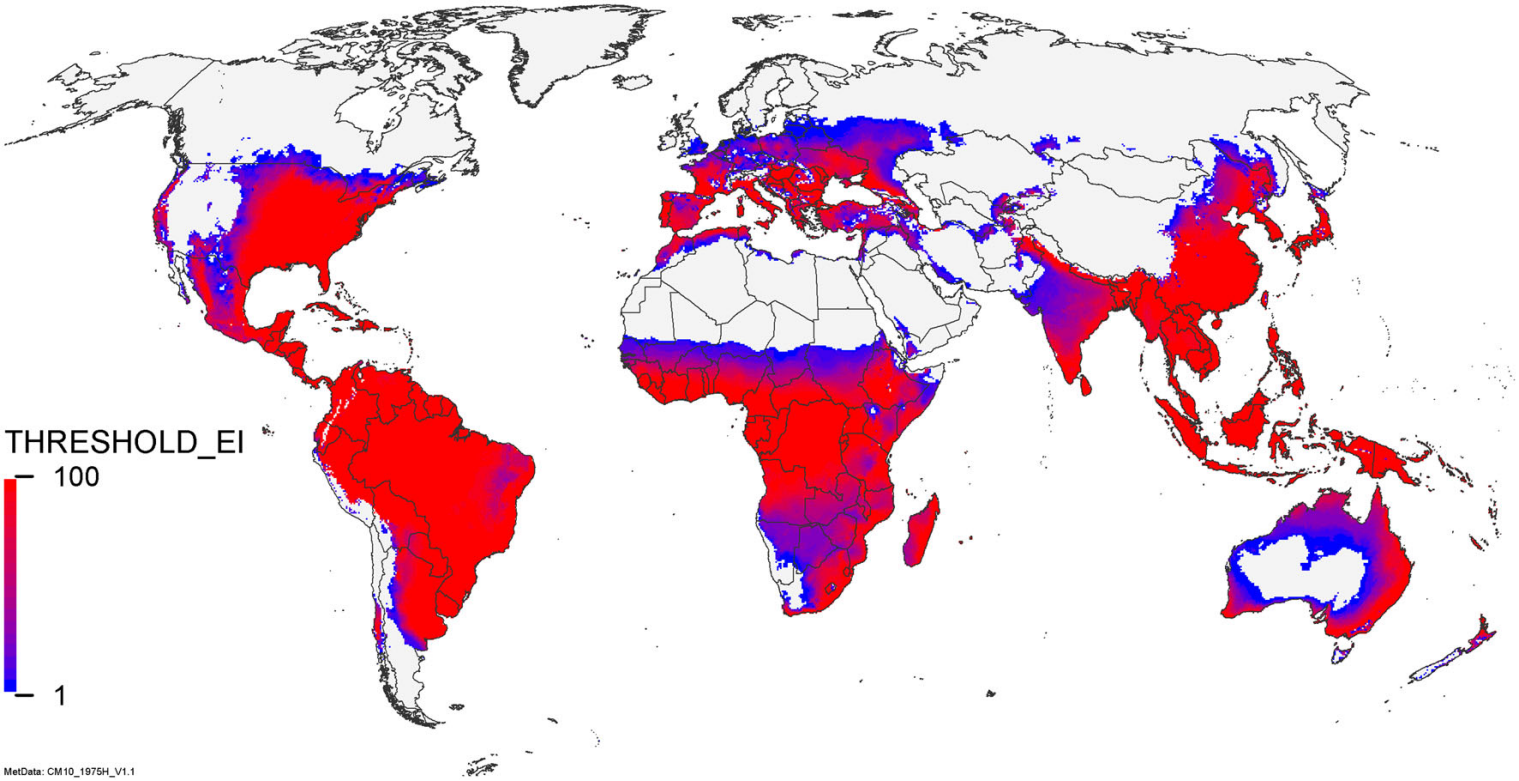
CLIMEX parametric uncertainty analysis. The proportional model agreement (%) for sampled parameter uncertainty

The model uncertainty is portrayed in Fig. 7. This map of model agreement for climate suitability for persistence indicates that there is a greater degree of geographical uncertainty in relation to the ability of *C. partellus* to persist in drier areas (e.g. sub-Sahelian Africa, Western India, Namibia, Central Australia) than colder areas (e.g. Northern China, Russia and Northern USA).

## Discussion

The re-fitted CLIMEX model we have produced highlights the substantial invasion risk posed by *C.* *partellus* to cropping regions in the Americas, Australia, China, Europe, New Zealand and West Africa. Its broad host range and reported impacts suggest that it should be a pest of significant concern to biosecurity agencies in these presently uninvaded regions, and particularly those countries adjacent to currently infested regions in Africa and Asia.

This CLIMEX model for *C. partellus* accords with the known distribution and other biological data substantially better than the three pre-existing models for the species. Sensitivity analyses suggest that there are no obvious concerns with sensitive parameters that are poorly understood, and the uncertainty map provides guidance regarding the geographical areas where we have greater or lesser confidence in the model performance.

The impact of a moderate amount of irrigation on the potential establishment range of *C. partellus* in Asia is clear in Fig. 3, as most of India and Pakistan increase in suitability. Under a natural rainfall scenario, the dry stress parameters are lethal in much of India and Pakistan where *C. partellus* occurs. However, *C.* *partellus* is a pest of the *kharif* (wet) season, and the model indicates that growth occurs during this season. As irrigation must be applied to support the *rabi* (dry season) crops, use of the irrigation scenario is justified to remove dry stress, making these areas suitable for *C. partellus*. This also fits with information on the use of irrigation in the Punjab region of Pakistan (Attique et al. 1980; Carl 1962), and along the Indus River to include Karachi and Hyderabad. With the irrigation scenario, growth charts for Hisar, India (Fig. 6b) accord well with the trapping results reported in Taneja and Leuschner (1985), and without irrigation, the model produces results for Jammu, in north-west India (Fig. 6a), which accord with the observations by Ahad et al. (2008).

In Africa, under a natural rainfall scenario, most locations, including some of the validation sites which are in relatively dry areas, are projected to be suitable for *C. partellus* (Fig. 4a). Other locations, found in the literature but not used to fit the model, are also projected to be suitable: Ethiopia (Assefa et al. 2010; Dejen et al. 2014), Kenya (Guofa et al. 2001), Lesotho (Ebenebe et al. 1999), Malawi (Paliani and Kapeya 2001), Mozambique (Cugala and Omwega 2001; Cugala et al. 2001; Moolman et al. 2014) and Zambia (Sohati et al. 2001).

Obopile and Mosinkie (2001) find *C. partellus* to be the most abundant and widely distributed stem borer in Botswana. Figure 1 in Obopile and Mosinkie (2001) largely corroborates the results of our model, with many location records falling in areas modelled as climatically suitable. Contentious areas include the Hukuntsi District (Kgalagadi North Region), possibly parts of the Bobonong District (Central Region), and Chobe, Ngamiland East and Ngamiland West in the Maun Region. Our model indicates all of these sites are too dry to sustain permanent populations, both under a natural rainfall scenario and in the composite irrigation map (Fig. 4a, b). However, they all have a positive GI_A_ even under a natural rainfall scenario (Fig. 4c), and hence could support seasonal populations. Under irrigation they are all modelled to be suitable, and our exploration of the Hukuntsi site (above) suggests that 2000 and 2001 were wetter than average years, and would have been suitable for the persistence of *C. partellus* during this time frame. Continued sampling throughout Botswana could assist in determining whether populations are seasonal or persistent, and whether or not the moisture and dry stress parameters in our model need to be adjusted.

Getu et al. (2001, 2003) and Assefa et al. (2010) note that *C. partellus* is absent from western Ethiopia, although this area is more suitable than the eastern part which borders with Somalia, and from where they think the parasitoid, *Cotesia flavipes*, came into Ethiopia. However, they do not indicate that *C. partellus* occurs anywhere near the border with Somalia: its distribution seems to be restricted to the more central regions of Ethiopia, which also happen to be modelled as the more suitable. It would be interesting to have more recent surveys to indicate whether or not *C.* *partellus* has expanded its range in this country.

According to Kfir (1988), under normal practices of weeding and overhead irrigation, *C. partellus* enters a diapause stage in the cold dry winter months in the Highveld region of South Africa. Our model shows that without irrigation, growth may not occur there from the end of May until the beginning of October, as it is too dry in this region. However, the winter months are still warm enough for growth to occur, albeit at a lower rate. When we look at the degree-days accumulated each week, and only begin to accumulate growth from October, as per the experiments of Kfir (1988) with planting done in October, we find that the first generation can be completed by the beginning of January, the second generation by mid-March, but the third generation is not completed until July, as degree-days are accumulated more slowly over the winter months. A fourth generation can be completed by October, when new crops are planted. This accords with the observation that moths appear as early as the beginning of September, well before new crops are planted, and that some of these can breed on sorghum shoots that sprout towards the end of winter (Kfir 1988, 1992). Kfir (1988) also notes that “*C. partellus* larvae begin to emerge from diapause during the second half of August…” when temperatures are increasing, but it is still too dry for growth unless irrigation is added, which suggests that larvae are not really in a full diapause state, just growing very slowly as a result of the cooler temperatures. This is supported by later results of (Kfir 1991), indicating that the availability of drinking water shortens the “diapause” period of *C. partellus*. If larvae are drinking water, they are presumably not in a true state of diapause. Various authors suggest that diapause in *C. partellus* is facultative, and the mechanisms inducing or terminating such a condition are as yet not understood (i.e. see Reddy in ICRISAT 1989; Kfir 1991, 1993). The fact that there are overlapping generations in southern Africa (Kfir 1988; Van Rensburg and Van den Berg 1992) that crops are grown with irrigation in the summer months (but it is not clear when this practice begins or ends), and that there appears to be a poorly understood diapause mechanism occurring (Kfir 1988; Van Rensburg and Van den Berg 1992) complicates the interpretation of the phenology observed in this area. Nonetheless, growth charts suggest that the model is correctly representing phenological patterns, and a sufficient number of generations are being simulated.

**Fig. 8:**
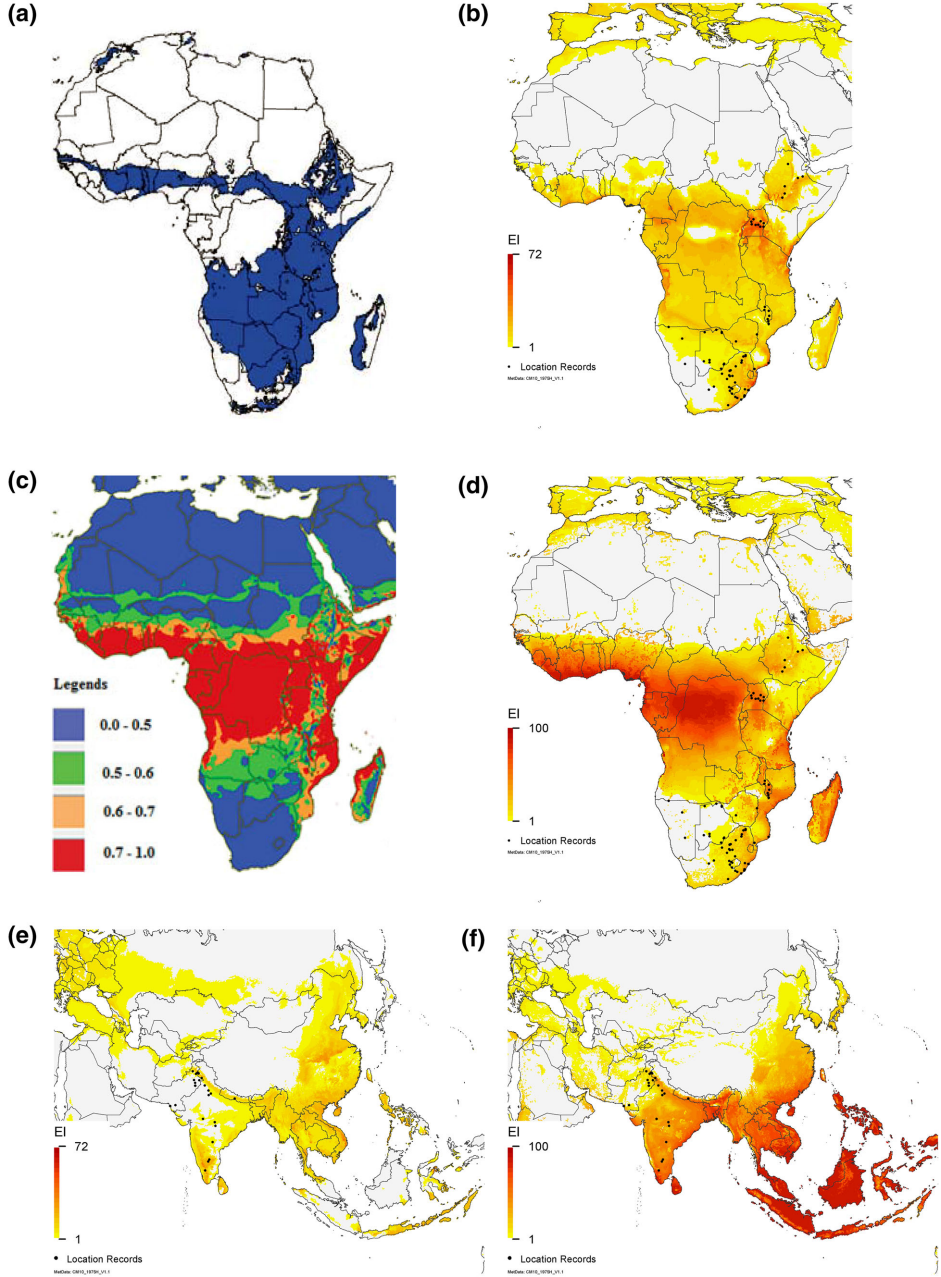
Distribution or climate suitability for *C. partellus *as modelled by **a** Overholt et al. (2000); **b**, **e** Hutchison et al. (2008); **c** Khadioli et al. (2014); (**d**, **f**) current CLIMEX model. Permission was obtained from both ICIPE and the Bulletin of Entomological Research to reproduce the Overholt et al. (2000) and Khadioli et al. (2014) figures

The modelled potential distribution of *C. partellus* in Africa is larger than the current known distribution, extending across the centre to western Africa, as with all previous published models for this species (Hutchison et al. 2008; Khadioli et al. 2014; Overholt et al. 2000) (Fig. 8). The projection of Overholt et al. (2000) is the most conservative, as it does not indicate that *C. partellus* could spread into central Africa (Democratic Republic of the Congo, Congo, Gabon, Equatorial Guinea), and it precludes persistence in Liberia and Sierra Leone (Fig. 8a). The two other models (Hutchison et al. 2008; Khadioli et al. 2014) (Figs. 8b, c respectively) suggest that *C. partellus* could persist right across central Africa, although the Hutchison et al. (2008) model suggests that the central part of the Democratic Republic of the Congo is unsuitable because it is generally too wet for growth (GI_A_ = 0). Nonetheless, along with the other models, our results highlight the fact that *C. partellus* may not have yet reached its potential distribution in Africa.

There are numerous differences between our CLIMEX model (Fig. 8d) and the GIS results of Overholt et al. (2000) (Fig. 8a). CLIMEX results in a slightly broader potential distribution in Kenya include all of Central Africa and most of the west coast countries as highly suitable; show all of Madagascar to be suitable, with the eastern seaboard more suitable than the seasonally drier west; exclude all of Namibia and much of Botswana as being too dry and show more of South Africa to be suitable, including those areas along the eastern coast where *C. partellus* occurs.

If we compare our results for Africa (Fig. 8d) to those of Hutchison et al.’s (2008) results (Fig. 8b), we increase the maximum suitability, alter where the most suitable regions for *C. partellus* are to be found, project Namibia and Botswana as being less suitable, and project an extension of the northern range limits. Our results for Asia are also significantly different (Fig. 8e vs. f): our model increases the degree of suitability in most of Asia, indicates that all of Southeast Asia is highly suitable and reduces suitability in the more northern (colder) regions.

When compared to the Khadioli et al. (2014) ILCYM model for Kenya (Fig. 8c), we find discrepancies in the projected distribution in Kenya: areas with a CLIMEX EI = 0 (Fig. 8d) have the highest establishment index (ERI) value, suggesting potential permanent establishment (Khadioli et al. 2014), and areas in southern and central Kenya designated as unsuitable by the ILCYM model (blue and green regions, Fig. 8c) are suitable in the CLIMEX model (Fig. 8d). Overall, the CLIMEX model (Fig. 8c) seems to distinguish between suitable areas somewhat better, as the ILCYM model shows the overwhelming majority of Kenya to have an ERI > 0.6 (or even >0.7) (Fig. 8c), and it does not preclude persistence in the very arid rangeland regions in the north-west of Kenya or near the border with Somalia, as does the CLIMEX model (Fig. 8d). For the rest of Africa, with the threshold ERI < 0.6 which Khadioli et al. (2014) use to designate areas as unsuitable for permanent establishment, the ILCYM model precludes the establishment of *C. partellus* in all of South Africa, Namibia, Botswana, Zimbabwe, southern Angola and most of Zambia (Fig. 8c), which is clearly at odds with many of the location records for this species. The curious north–south banding across the southern Saharan Desert border in Khadioli et al. (2014) is surely a modelling artefact.

Thus, each of the four models (Hutchison et al. 2008; Khadioli et al. 2014; Overholt et al. 2000, and ours) provides somewhat different results to the others, and each suffers from some deficiency. The GIS model of Overholt et al. (2000) only considers a limited set of meteorological data (mean values for maximum temperature, evapotranspiration, precipitation and elevation), combined in a somewhat simplistic manner. This could result in locations with quite different climates showing up as suitable: a location with a relatively constant climate could have the same mean values as one with a greater range of values, potentially explaining some of the acknowledged errors in these results (over-estimates risk in Zimbabwe, under-estimates it in South Africa). The CLIMEX model of Hutchison et al. (2008) contains an intrinsic error, since dry stress accumulates within the soil moisture range suitable for growth, and some of the other parameter values (e.g. lower and upper thresholds for development) are not consistent with values found in the literature. The ILCYM model of Khadioli et al. (2014) is a temperature-driven model, ignoring all other factors that may influence the life cycle of a species, and the suitability of an area for persistence, thereby excluding persistence of *C. partellus* from known suitable locations in South Africa, Botswana and Zimbabwe. Our CLIMEX model may suffer from some parameter estimations in the absence of robust information (in particular, PDD), but the values we have used are all biologically plausible, and we have considered both temperature and soil moisture conditions conducive to population growth and persistence as well as a variety of stress mechanisms that could preclude persistence in different areas for different reasons (i.e. heat stress vs. cold stress vs. dry stress). However, despite their differences, all of the models indicate that *C. partellus* can occupy a much larger range in Africa than it currently does, and has the potential to expand into West Africa.

For this study, we examine more closely our understanding of the pest risk of *C. partellus* by considering in the first instance the use of irrigation and including this scenario in our maps only for those areas designated as using irrigation (Siebert et al. 2005) (Figs. 3b,4b and 5a), and in the second instance, by then overlaying these areas onto areas where cropping of host plants (maize, sorghum, sugarcane, rice and pearl millet) occurs, using the Spatial Allocation Models (MapSPAM) (You et al. 2012, 2014) (Figs. 3b, c, 4b–d, and 5). There were several issues associated with this, which we have attempted to resolve. The first problem we encountered was a difference in the areas lacking agricultural production data in the two versions of MapSPAM. We were only able to obtain a comprehensive picture of agriculture by combining the data from the two versions (You et al. 2012, 2014), to produce a map layer indicative of where these crops were known to have been grown in recent history. Secondly, whilst most agricultural areas globally are indicated as being suitable for seasonal growth with irrigation, there are still some regions in Asia (i.e. along the north of Nepal and Bhutan) that show up as having host crops, but appear unsuitable for growth of *C. partellus* (Figs. 3c, 5b). Given the climatic conditions of these locations, with very low maximum temperatures and high altitudes, it is unlikely that the host crops are actually grown there. This is more likely to be an error in the way that agricultural data have been allocated to a region, and we can discount these areas as being able to sustain host crops completely free from any risk of attack by *C. partellus*. Thus, although we have possibly overestimated the cropping area by using a union of the two versions of MapSPAM (You et al. 2012, 2014), we are nonetheless able to provide a reasonable assessment of the risk of pest attack to host crops—either due to the occurrence of persistent, permanent populations of *C.* *partellus*, or as a result of seasonal incursions—and we have been able to explain apparent discrepancies in the results.

The significant differences between the published models for *C. partellus* highlight some of the challenges in crafting reliable pest risk models. Good pest risk models benefit from access to quality distribution data and careful consideration of relevant eco-physiological information and field observations. These sources of information are often contradictory and require a process of careful scrutiny, perhaps applying Chamberlin’s method of multiple competing hypotheses (Chamberlin 1890). Including consideration of non-climatic range-limiting factors such as crop distribution and irrigation allows the model to be framed using biologically meaningful parameters. To ignore the importance of such non-climatic factors can lead to model distortions. For example, ignoring the effects of irrigation could have resulted in the model being fitted with unrealistically low thresholds of soil moisture for growth, and consequently over-estimating the potential risks into xeric regions. Explicitly incorporating such non-climatic factors also enables the analyst to understand how risks can be affected by factors such as agricultural expansion or implementation of irrigation, and therefore how to better manage these risks.

## Author contributions

TY did the literature survey, fitted the model and wrote the manuscript. DJK developed the composite irrigation method, assisted in parameter fitting and writing the manuscript. NO generated shapefiles used in the analyses and created all maps. JVdB provided location records and information on African locations. WDH provided the parameter values of the Hutchison et al. (2008) model and location records. All authors read, modified and approved the manuscript.
